# Anti-M: Report of two cases and review of literature

**DOI:** 10.4103/0973-6247.42695

**Published:** 2008-07

**Authors:** Rashmi Tondon, Rahul Kataria, Rajendra Chaudhry

**Affiliations:** *Department of Transfusion Medicine, Sanjay Gandhi Post Graduate Institute of Medical Sciences, Rae Bareily Road, Lucknow, Uttar Pradesh, India*

**Keywords:** Anti-M, immunizing, naturally occurring

## Abstract

Anti-M is a fairly common naturally occurring antibody with rarely causing hemolytic transfusion reactions or hemolytic disease of the newborn. Most anti-M are not active at 37°C and can generally be ignored in transfusion practice. However, we did not find this antibody to be fairly common and detected only two cases of anti-M in the past three years. We describe these two cases; one ‘immunizing’ type and other ‘naturally occurring’ and review the literature. The immunizing type was reactive at 37°C as well as AHG phase of testing with IgG component, and showing dosage effect while the other was ‘naturally occurring’ reactive well below 37°C. Though rare, sometimes these antibodies can be of clinical significance when the antibody detected is reactive at 37°C and AHG phase. When the antibody is active at 37°C, M antigen negative cross match compatible red cell unit should be given.

## Introduction

Anti-M is a fairly common naturally occurring antibody[1,2] originally described by Wolff and Johnsson in 1933.[3] Only rarely it has been implicated as a cause of immediate and delayed[4–5] hemolytic transfusion reactions or hemolytic disease of the newborn.[6] Though it is the frequently encountered antibody of the MNSs blood group system, it is not considered to be clinically significant. Most Anti-M is only reactive at temperatures below 37°C with an optimum temperature of 4°C, though occasional examples will agglutinate red cells at 37°C. It appears to be more common in infants than in adults. Our experience with regard to anti-M has been different and in the past three years, we did not find this antibody to be ‘fairly common’ and encountered with only two cases of anti-M during this period. Both cases were referred to the Immunohematology laboratory from the Cross match laboratory, one presenting as Cross match incompatibility and other as blood group discrepancy.

## Case Reports

### Case 1

**Figure 1:**
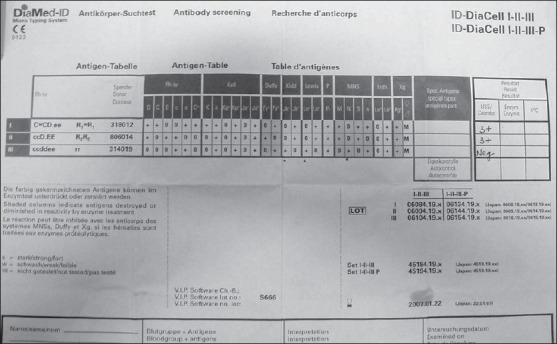
Antigram of screening 3 cell panel

**Figure 2:**
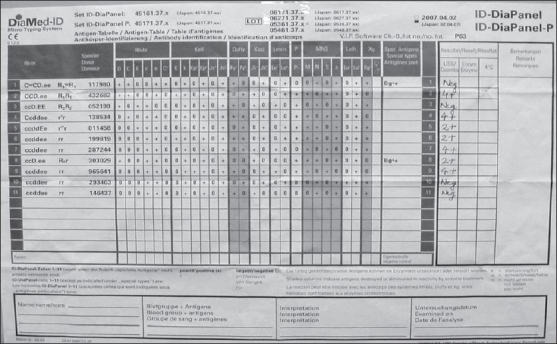
Antigram of 11 cell panel used in antibody identification

A 32-year-old multiparous female, with serous cystadenoma, pancreas presented with signs and symptoms of anemia with hemoglobin – 5.8 g/dl and hematocrit – 17.4% and microcytic hypochromic picture on peripheral blood smear. Three units of packed red cells were requested for transfusion. The blood group of the patient was typed as A Rh (D) +ve. However, all the donor red cell units were incompatible by Indirect Antiglobulin Test (IAT) with both gel technique (Diamed-ID Microtyping system) and conventional test tube method. The sample was referred to the Immunohematology lab for workup. Direct antiglobulin test (DAT) was performed on red cells from EDTA - anticoagulated samples using polyspecific antiglobulin reagents (anti IgG and C3d) but was found to be negative along with negative autocontrol. Antibody screening procedure involved indirect antiglobulin test with antihuman globulin (AHG) using low-ionic strength solution (LISS - IAT). LISS - IAT screening test with commercially available three cell panel (Diamed-ID Microtyping system) showed positive reactions with panel I and II (3+) while negative with III panel cells [Figure 1]. The suspected antibody was positive at all phases of testing. The antibodies that were kept in the differential were anti-D, Jk^b^, Le^b^, M, and S. Antibody showed the probable pattern as anti-M using 11 identification cell panel which was 4+ positive with homozygous M+M+ cells (panel 2, 4, 7, 9) and 2+ reaction with heterozygous M+ N+ cells (panel 5, 6 and 8) and negative with M - cells in the panel 1, 3, 10, 11 [Figure 2]. However, the possibility of anti-S could not be clearly ruled out. Confirmation of antibody as anti-M was further performed in two ways. Firstly, three ‘O’ red cells positive for M antigen i.e. homozygous MM red cells and negative for S antigen while three negative units for M antigen (M-N+) and positive for S antigen were cross matched with the patient‘s serum which showed reaction with units positive for M antigen while it was negative with units negative for this antigen. The possibility of anti-S was ruled as the negative reaction was observed with S antigen positive cells. Secondly, this antibody failed to react with papain premodified red cells.

In order to determine the Immunoglobulin class of antibody, titration studies of the sera was done before and after treatment with dithiothreitol.[7] The antibody persisted after the serum was treated with dithiothreitol suggesting the presence of IgG component along with IgM. Red cell phenotyping of the patient was negative for M antigen. Thus, anti-M found in the female patient with IgG component, reactive at 37°C as well as AHG phase of testing and showing dosage effect could probably have been of potential significance. The patient was successfully transfused with M negative red blood cells.

### Case 2

This case was detected in a 15-year-old male patient with diagnosis of systemic lupus erythromatosus (SLE) with lupus nephritis-class 4. Laboratory parameters showed low hemoglobin value of 9.9 g/dl with reticulocyte count of 6% though there was no clinical evidence of hemolysis. The case presented in the cross-match lab as blood group discrepancy with forward grouping showed B Rh(D)+ve while reverse grouping showed panreactivity (2+) with all three cells (A,B,O cells). Thinking that we are probably dealing with a case of autoimmune hemolytic anemia secondary to SLE, the case was referred to Immunohematology lab for further work-up. The discrepancy in reverse grouping was resolved when the test tubes were incubated at room temperature (22° C), 37°C and at 4°C. There was panreaction at 4°C and 22°C but disappeared at 37°C showing B group and indicating that the antibody is reactive below 37°C. DAT and Autocontrol were negative by both gel technique and conventional tube methods ruling out the obvious possibility of autoantibody. LISS - IAT showed 4+ reaction with panel I and II and negative with panel III. With conventional tube technique using strict prewarmed conditions for 37°C and AHG phase testing, the antibody was reactive at 4°C and at room temperature with reaction eliminating at 37°C. The antibody was well identified as anti-M by the 11 cell identification panel. The A and B cells negative for M antigen were used for reverse typing and the results clearly indicated B group of the patient as well as supporting the fact that the unexpected alloantibody causing discrepancy was anti-M. As there was no history of transfusion, anti-M detected was labeled as ‘naturally occurring’. Immunoglobulin class identified in this case by Dithiothreitol treatment suggested only IgM component in the patient’s serum. Though anti-M detected was reactive well below 37°C, the patient was still transfused M negative red cells.

## Discussion

Most anti-M are not active at 37°C, they can generally be ignored in transfusion practice. However, when anti-M is active at 37°C, M antigen negative cross match compatible red cell unit should be given. This holds true for case 1 where the antibody detected was reactive at 37°C and AHG phase with partial IgG component and therefore can be considered of potential clinical significance. Clinically insignificant anti-M that reacts strongly at room temperature and not at all at 37°C could be misinterpreted as 37°C reactive if the reactants in the test tube are permitted to cool after centrifugation prior to evaluating hemagglutination reactions. Furthermore, certain anti-M antibodies with high titer and high affinity such as anti-M may react strongly at room temperature and cause hemagglutination to carry through 37°C and the antiglobulin test phase. This may be a possible reason for showing panreaction with the screening cells panel where strict warm conditions were not maintained during centrifugation. Therefore to rule out the possible reactivity observed at the AHG phase is due to the presence of IgG and not because of the binding of IgM from the room temperature test phase, testing should be done in strict warm conditions.

Though majority of anti-M antibodies are IgM antibodies and are non-complement activating,[8] however, IgG component may coexist alone or in combination which may be complement activating. Freedman *et al*, in 1980[9] described the complement activating nature of anti-M by testing with ^125^ I- labeled anti C3d. Combs *et al*, in 1991[10] described an autoanti-M causing hemolysis *in vitro* by activating complement in conventional tube testing at low ionic strength. At least 15 cases of patients with autoanti-M have been reported and reviewed by Sacher *et al*.[11] According to him, 11 were non significant while rest four gave some symptoms of cold hemagglutinin disease. Anti–M may show a dosage effect as in the case of the female patient, reacting more strongly with homozygous cells (M+N-) than cells (M+N+).[12] Incidence of anti-M in donor sera was found be 1 in 2500 when reacting with homozygous M+N- cells while the incidence reduced to half i.e.1 in 5000 when screened with heterozygous M+N+ cells indicating that some weaker examples of anti-M may be missed with heterozygous cells.

MN antibodies are often pH dependent. IgM anti-M has an optimum pH of 6.5 and are mostly inactive at pH 7.5, and below pH 6.5, they become non-specific.[13] Another feature of this antibody is its failure to react with ficin or papain premodified cells. Proteolytic enzymes, such as ficin or papain, cleave red cell membrane sialoglycoproteins at well defined sites. Reactivity of anti-M is abolished by commonly used enzyme techniques.[14] The effect of enzyme on the expression of MNSs system antigens reflect the point at which the particular enzyme cleaves the antigen bearing sialoglycoprotein and the position of antigen relative to the cleavage site. Rarely anti-M has been implicated in immediate and delayed hemolytic transfusion reactions which are supported by the results of ^51^Cr survival tests and monocyte phagocytosis assays.[15] These examples demonstrate that anti-M can at times be of clinical importance and interpretation of test results should be done with caution.

Hemolytic disease of newborn (HDN) of varying degrees of severity has been reported in association with anti-M and can even lead to intrauterine deaths[16] or requiring treatment by exchange.[17] Detection of anti-M in antenatal screening for antibodies as described in the literature varies from second commonest non-Rh antibody after anti- Kell[18] to a rare finding.[19,20] One high titer IgG plus IgM anti-M was responsible for neonatal red cell aplasia and caused a substantial reduction in proliferation of erythroid cells in culture.[21] Therefore, like anti-K, anti-M may cause HDN primarily by destroying erythroid progenitors rather than mature erythrocytes. Sources of anti-M blood grouping reagent can be quite different varying from human anti-M, rabbit anti-M to monoclonal anti-M. Though a seed extract of Iberis Amara was found to have specificity[22] but no seed lectin has proved satisfactory as an anti-M blood grouping reagent.[23] Because of the different sources, manufacturer's directions are quite different and one must be aware of these differences. In addition, because of the varied sources of reagents and the segments of glycoprotein's detected, results may be different when different types of reagents are used.

Clinically insignificant anti-M that reacts strongly at room temperature and not at all at 37°C could be misinterpreted as 37°C reactive if the reactants in the test tube are permitted to cool after centrifugation prior to evaluating hemagglutination reactions. Furthermore, certain anti-M antibodies with high titer and high affinity such as anti-M may react strongly at room temperature and cause hemagglutination to carry through 37°C and the antiglobulin test phase. Therefore to rule out the possible reactivity observed at the AHG phase is due to the presence of IgG and not binding of IgM from the room temperature test phase, testing should be done in strict warm conditions. Thus, these case reports on anti-M indicate how this antibody can have varied presentations. Though rare, sometimes these antibodies can be of clinical significance when the antibody detected is reactive at 37°C and AHG phase with partial IgG component. ‘Naturally occurring’ antibody can usually be ignored especially when antibody is reactive well below 37°C. Strict warm testing conditions must be followed to rule out false positive reactions.
